# Deep Learning Approaches for Medical Image Analysis and Diagnosis

**DOI:** 10.7759/cureus.59507

**Published:** 2024-05-02

**Authors:** Gopal Kumar Thakur, Abhishek Thakur, Shridhar Kulkarni, Naseebia Khan, Shahnawaz Khan

**Affiliations:** 1 Department of Data Sciences, Harrisburg University of Science and Technology, Harrisburg, USA; 2 Department of Computer Application, Bundelkhand University, Jhansi, IND

**Keywords:** clinical practice, medical imaging, reliability, machine learning, artificial intelligence

## Abstract

In addition to enhancing diagnostic accuracy, deep learning techniques offer the potential to streamline workflows, reduce interpretation time, and ultimately improve patient outcomes. The scalability and adaptability of deep learning algorithms enable their deployment across diverse clinical settings, ranging from radiology departments to point-of-care facilities. Furthermore, ongoing research efforts focus on addressing the challenges of data heterogeneity, model interpretability, and regulatory compliance, paving the way for seamless integration of deep learning solutions into routine clinical practice. As the field continues to evolve, collaborations between clinicians, data scientists, and industry stakeholders will be paramount in harnessing the full potential of deep learning for advancing medical image analysis and diagnosis.

Furthermore, the integration of deep learning algorithms with other technologies, including natural language processing and computer vision, may foster multimodal medical data analysis and clinical decision support systems to improve patient care. The future of deep learning in medical image analysis and diagnosis is promising. With each success and advancement, this technology is getting closer to being leveraged for medical purposes. Beyond medical image analysis, patient care pathways like multimodal imaging, imaging genomics, and intelligent operating rooms or intensive care units can benefit from deep learning models.

## Introduction and background

Medical imaging plays a critical role in modern healthcare, enabling clinicians to visualize internal structures, detect abnormalities, and guide treatment decisions [1,2]. Modalities such as magnetic resonance imaging (MRI), computed tomography (CT), X-rays, and ultrasound produce vast amounts of data that necessitate efficient and accurate analysis [3]. Traditionally, image analysis methods have relied on handcrafted features and heuristic algorithms, which often struggle to fully capture the complexity and variability inherent in medical images [4].

The emergence of deep learning, particularly convolutional neural networks (CNNs), has revolutionized medical image analysis by offering a paradigm shift in how features are extracted, and representations are learned. Unlike traditional methods that require manual feature engineering, deep learning algorithms can automatically learn hierarchical representations directly from raw data. This capability has proven to be particularly advantageous in medical imaging, where images can be high-dimensional, noisy, and exhibit subtle patterns or abnormalities [5].

By leveraging CNNs, deep learning models can discern intricate patterns and relationships within medical images, leading to improved accuracy and efficiency in tasks such as classification, segmentation, detection, and reconstruction. The ability of deep learning algorithms to adapt and generalize from diverse datasets further enhances their utility across various imaging modalities and clinical applications [6].

## Review

Deep learning architectures for medical image analysis

Medical image analysis, a crucial component of modern healthcare, relies heavily on advanced computational techniques to extract meaningful information from the imaging data. Deep learning, particularly CNNs and recurrent neural networks (RNNs), has emerged as a powerful tool in this domain, offering unprecedented capabilities for automated feature extraction, pattern recognition, and decision-making. This section explores the landscape of deep learning architectures tailored for medical image analysis, highlighting their design principles, applications, and impact on clinical practice [7].

Convolutional neural networks (CNNs)

CNNs have revolutionized various fields, including computer vision and medical imaging, owing to their ability to directly learn hierarchical representations from raw pixel data. In medical image analysis, CNNs serve as the cornerstone for tasks such as image classification, segmentation, detection, and reconstruction [8]. One of the most prominent architectures in medical image segmentation was U-Net, introduced by Ronneberger et al. in 2015 [9]. The U-Net architecture comprises a contracting path, which captures contextual information through successive convolutional and pooling layers, followed by an expansive path that enables the precise localization of objects of interest. This symmetric architecture allows for efficient segmentation of structures while preserving spatial details, making it well suited for tasks such as tumor delineation in MRI scans or organ segmentation in CT images [9]. In addition to segmentation, CNNs have demonstrated remarkable success in various other medical imaging tasks. For instance, in radiology, CNNs have been employed for automated detection and classification of abnormalities in chest X-rays [2]. Additionally, CNN-based models have shown promise for tasks such as lesion detection in mammograms, retinal vessel segmentation in fundus images, and brain tumor segmentation in MRI scans [10,11].

Recurrent neural networks (RNNs)

While CNNs excel at processing spatial data, RNNs are specifically designed for sequential data analysis, making them well-suited for tasks involving temporal dependencies [12]. In medical imaging, RNNs and their variants, such as long short-term memory (LSTM) networks, have been used to analyze time-series data and sequential imaging modalities [12]. One notable application of RNNs in medical image analysis is the interpretation of electrocardiograms (ECG) for the detection of cardiac arrhythmia. Hannun et al. demonstrated the efficacy of a deep neural network in achieving cardiologist-level performance in arrhythmia detection from ambulatory electrocardiogram recordings [13]. By effectively capturing the temporal dependencies and subtle patterns in echo signals, LSTM-based models can aid clinicians in diagnosing cardiac abnormalities with high accuracy and reliability.

Although CNNs and RNNs serve as foundational architectures for medical image analysis, researchers continue to innovate by developing customized architectures tailored to specific clinical applications and imaging modalities. For instance, attention mechanisms that enable models to focus on relevant regions of interest within an image have been integrated into CNNs for tasks such as pathology image analysis and anomaly detection in medical images [4]. Future research directions in deep learning for medical image analysis are poised to address several key challenges and opportunities. Efforts are underway to enhance the interpretability and explainability of deep learning models, facilitating their adoption in clinical settings where transparency and trust are paramount. Moreover, advancements in multimodal learning, federated learning, and transfer learning hold promise for leveraging complementary information from diverse imaging modalities and datasets, thereby improving the robustness and generalization capabilities of deep learning models [14].

Table 1 provides a concise summary of the discussed deep learning architectures, their respective applications in medical image analysis, and their overall impact on healthcare delivery.

**Table 1:** Summary of deep learning architectures and their impact on medical image analysis in radiology, oncology, and pathology MRI: Magnetic resonance imaging; GNNs: Graph neural networks; ECG: Electrocardiogram The table is the authors' own creation.

Architecture	Applications	Impact on Medical Image Analysis
Convolutional Neural Networks (CNNs)	Automated detection and classification of abnormalities in chest X-rays, lesion detection in mammograms, retinal vessel segmentation in fundus images, brain tumor segmentation in MRI scans	Revolutionized radiological practices by expediting diagnosis, reducing workload, and improving patient outcomes
Recurrent Neural Networks (RNNs)	Interpretation of ECG for cardiac arrhythmia detection, analysis of time-series data in oncology imaging	Facilitated accurate detection of cardiac abnormalities and longitudinal analysis of treatment response
Customized Architectures	Integration of attention mechanisms into CNNs for pathology image analysis, development of advanced architectures such as GNNs	Enhanced model performance, interpretability, and generalization capabilities in medical image analysis

Applications in medical image analysis

The application of deep learning techniques in medical image analysis has witnessed significant advancements, revolutionizing various clinical domains and imaging modalities. From radiology to oncology and pathology, deep learning algorithms have demonstrated remarkable efficacy in automating tasks, improving diagnostic accuracy, and facilitating clinical decision-making.

Deep learning in radiology

In the field of radiology, the integration of deep learning algorithms has ushered in a new era of automated detection and classification of abnormalities in chest X-rays, a widely used imaging modality for diagnosing pulmonary diseases, cardiac conditions, and thoracic injuries [15]. This section discusses the transformative impact of CNNs in revolutionizing radiological practices, highlighting their capabilities, applications, and implications for clinical workflows [15].

Automated detection and classification

CNNs, which are renowned for their advantages in learning intricate patterns directly from pixel data, have been instrumental in automating the detection and classification of abnormalities in chest X-rays [16]. These deep learning models have exhibited remarkable performance in identifying various pathologies, including pulmonary nodules, pneumothorax, and pneumonia, with high sensitivity and specificity [17]. For instance, researchers have developed CNN architectures specifically tailored for detecting signs of pneumonia on chest X-rays, enabling early diagnosis and prompt initiation of treatment. By analyzing pixel-level features and spatial relationships within the images, CNN-based models can accurately pinpoint areas indicative of pathology, thereby assisting radiologists in their diagnostic endeavors [18]. CNNs have also been deployed for the classification of chest X-rays based on the presence or absence of specific pathologies. By utilizing large-scale annotated datasets and sophisticated network architectures, these models can categorize images into different diagnostic categories, streamlining the interpretation process and alleviating the workload burden on radiologists [19].

Clinical impact and implications

The integration of deep learning algorithms into radiological workflows holds immense promise for improving patient care and enhancing diagnostic accuracy. By automating the detection of abnormalities in chest X-rays, CNN-based models can expedite the diagnostic process, leading to earlier detection, timely intervention, and improved patient outcomes [20]. Furthermore, the deployment of deep learning models for image classification can aid radiologists in triaging cases, prioritizing high-risk patients, and facilitating efficient resource allocation. By providing clinicians with augmented decision support tools, these models empower radiologists to make more informed and confident diagnostic decisions, ultimately enhancing the quality and efficiency of healthcare delivery [21]. However, the widespread adoption of deep learning in radiology presents several challenges. Issues related to model interpretability, reliability, and generalization across diverse patient populations and imaging settings must be carefully addressed to ensure the safe and effective integration of these algorithms into clinical practice [4,22].

Future directions and research opportunities

Looking ahead, future research endeavors in deep learning for radiology are poised to explore new frontiers and address existing challenges. Efforts to enhance model interpretability, explainability, and robustness are critical for fostering trust and acceptance among radiologists and healthcare providers. Moreover, advancements in multimodal learning, transfer learning, and federated learning hold promise for leveraging complementary information from diverse imaging modalities and datasets, thereby improving the performance and generalization capabilities of deep learning models [23]. To summarize, the integration of deep learning algorithms, particularly CNNs, into radiological practices represents a paradigm shift in medical imaging, offering unprecedented capabilities in the automated detection and classification of abnormalities in chest X-rays. While challenges remain, the transformative impact of deep learning on radiology holds immense potential for improving patient care, enhancing diagnostic accuracy, and revolutionizing healthcare delivery in the years to come.

Oncology

In oncology, deep learning techniques have been extensively utilized for various tasks including tumor detection, segmentation, and treatment response assessment, particularly in MRI and CT scans. These imaging modalities play crucial roles in cancer diagnosis, staging, and treatment planning [24]. Deep learning models trained on large datasets of annotated medical images have demonstrated remarkable performance in accurately localizing and delineating tumors from the surrounding tissues. For instance, CNN-based architectures have been employed for brain tumor segmentation in MRI scans, enabling precise delineation of tumor boundaries and aiding in surgical planning and radiation therapy [25]. Similarly, deep learning algorithms have been applied to CT scans for the detection and characterization of lung nodules, which is a critical task in early lung cancer diagnosis and prognosis [26]. Furthermore, deep learning-based approaches have been instrumental in assessing treatment response and monitoring disease progression in oncology. By analyzing longitudinal imaging data, these algorithms can quantitatively evaluate changes in tumor size, shape, and enhancement patterns following treatment, thereby providing valuable insights into treatment efficacy and patient outcomes [27].

Pathology

In pathology, where the examination of tissue specimens plays a central role in cancer diagnosis and grading, deep learning techniques have emerged as powerful tools for automating image analysis tasks. Pathological image analysis often involves the interpretation of histological slides stained with various dyes to visualize cellular structures and tissue morphology. Deep learning models, particularly CNNs, have been trained on large datasets of annotated pathology images to perform tasks such as cancer diagnosis, grading, and prognostication. These models can accurately identify and classify cancerous cells, distinguish between different histological subtypes, and predict patient prognosis based on tissue morphology and biomarker expression patterns [28,29]. Moreover, deep learning-based approaches have facilitated the development of computer-aided diagnosis (CAD) systems in pathology, where algorithms assist pathologists in interpreting complex histopathological images and making accurate diagnostic decisions. These CAD systems have the potential to improve diagnostic accuracy, reduce inter-observer variability, and enhance workflow efficiency in pathology laboratories [30].

Table 2 summarizes the diverse applications of deep learning in radiology, oncology, and pathology.

**Table 2:** Deep learning applications in medical imaging domains CNNs: Convolutional neural networks; MRI: Magnetic resonance imaging; CT: Computed tomography The table is the authors' own creation.

Domain	Application	Description	Impact
Radiology	Automated detection of abnormalities	Utilizes CNNs to automatically detect abnormalities such as pulmonary nodules, pneumothorax, and pneumonia in chest X-rays	Accelerates diagnosis, reduces the workload for radiologists, enables early detection of diseases
	Classification of chest X-rays	Employs CNN-based models to classify chest X-rays based on the presence or absence of specific pathologies, aiding in triaging and prioritizing cases	Streamlines interpretation process, facilitates efficient resource allocation, improves diagnostic accuracy
Oncology	Tumor detection and segmentation	Utilizes CNN-based models to detect and segment tumors in MRI and CT scans, enabling precise delineation of tumor boundaries for treatment planning	Improves treatment planning, facilitates accurate tumor localization, enhances patient outcomes
	Treatment response assessment	Analyzes longitudinal imaging data using deep learning algorithms to evaluate treatment response and monitor disease progression in oncology patients	Provides valuable insights into treatment efficacy, enables personalized treatment strategies, enhances patient care
Pathology	Cancer diagnosis and grading	Employs CNNs to analyze histopathological images and perform tasks, such as cancer diagnosis, grading, and prognostication, based on tissue morphology and biomarker expression	Improves diagnostic accuracy, reduces inter-observer variability, enables more precise prognostication
	Computer-aided diagnosis (CAD)	Develops CAD systems using deep learning algorithms to assist pathologists in interpreting histopathological images and making accurate diagnostic decisions	Enhances workflow efficiency, reduces diagnostic errors, improves consistency in diagnostic interpretations

Challenges and limitations in deep learning for medical image analysis

Deep learning techniques have demonstrated remarkable success in various medical image analysis tasks ranging from segmentation to classification and diagnosis. However, despite their promising performance, these approaches face several challenges and limitations that must be addressed to realize their full potential in clinical practice. One of the primary challenges faced by deep learning approaches in medical image analysis is the requirement for large annotated datasets. Deep learning algorithms rely on labeled data for training, wherein each image must be annotated with ground-truth information, such as pixel-level segmentation masks or disease labels [31]. However, obtaining such annotated datasets in the medical domain can be labor-intensive, time-consuming, and expensive [4,10]. Moreover, the quality and consistency of annotations can vary across datasets, leading to potential biases and inaccuracies in the model training. Annotated medical datasets may also be limited in size, particularly for rare diseases or specific patient populations, hindering the development and evaluation of robust deep learning models [4]. Addressing this challenge requires collaborative efforts among healthcare institutions, research organizations, and data scientists to curate large-scale annotated datasets and establish standardized protocols for data annotation and sharing. Furthermore, techniques such as data augmentation, transfer learning, and semi-supervised learning can be employed to effectively leverage limited annotated data and improve the model performance.

Ensuring the robustness and interpretability of deep learning models is another significant challenge in medical image analysis, particularly in critical applications in which transparency and trust are essential. Deep-learning models are often regarded as "black-box" systems, wherein the internal mechanisms governing their decisions are opaque and difficult to interpret [4,32]. In healthcare, understanding how a model arrives at a particular diagnosis or decision is crucial for its clinical acceptance and adoption. However, the complex and nonlinear nature of deep learning architectures makes it challenging to interpret their underlying features and decision-making processes [33]. Moreover, deep learning models may exhibit vulnerabilities to adversarial attacks, wherein small imperceptible perturbations to input images can lead to erroneous predictions. These vulnerabilities pose serious concerns in medical imaging applications where misdiagnosis or erroneous predictions can have severe consequences for patient safety and well-being [32].

Addressing these challenges requires the development of techniques and methodologies to enhance the interpretability, robustness, and reliability of deep learning models for medical image analysis. Explainable AI (XAI) techniques such as attention mechanisms, saliency maps, and gradient-based visualization methods can help elucidate the rationale behind model predictions and improve transparency [34]. In addition, robust training strategies, regularization techniques, and adversarial defense mechanisms can enhance the resilience of deep learning models against adversarial attacks and improve their generalization capabilities across diverse patient populations and imaging protocols [32].

Another challenge in deep learning for medical image analysis is the generalization of the models across different patient populations and imaging protocols. Medical imaging datasets often exhibit inherent heterogeneity owing to variations in patient demographics, imaging modalities, acquisition protocols, and hardware settings [4]. Deep learning models trained on data from one population or imaging center may struggle to generalize to unseen data from different populations or acquisition protocols, leading to performance degradation and reduced reliability in real-world clinical settings. This challenge is exacerbated by the lack of standardized imaging protocols and variability in imaging quality across healthcare institutions [35].

To address this challenge, researchers are exploring techniques for domain adaptation, transfer learning, and multicenter collaboration to improve the generalization capabilities of deep learning models across diverse datasets and imaging settings. By leveraging techniques such as domain-specific normalization, feature alignment, and adversarial training, deep learning models can learn robust and transferable representations that are less sensitive to domain shifts and variations in the imaging data.

Future directions in medical image analysis

As deep learning continues to revolutionize medical image analysis, the field is poised for further advancements that address current challenges and leverage emerging opportunities. Interdisciplinary collaboration among clinicians, data scientists, and domain experts is essential to drive innovation and translate research findings into clinical practice.

One of the primary challenges in medical image analysis is the scarcity of large annotated datasets, which are essential for effectively training deep learning models. Addressing this challenge requires developing techniques for efficient transfer learning and domain adaptation. Transfer learning involves pre-training deep learning models on large datasets from related tasks or domains and fine-tuning them on smaller, task-specific datasets. By leveraging knowledge transfer from pre-trained models, transfer learning can significantly reduce the need for extensive annotated data and accelerate model development [4,36].

Moreover, domain adaptation techniques aim to adapt deep learning models to new imaging modalities or clinical settings, where labeled data may be limited or unavailable. By learning domain-invariant representations from the source and target domains, domain adaptation algorithms enable effective knowledge transfer and generalization across different data distributions. Future research may focus on advancing transfer learning and domain adaptation methods tailored to the specific challenges and requirements of medical image analysis tasks [37].

Medical imaging often involves the integration of information from multiple imaging modalities to obtain a comprehensive understanding of the underlying pathology. Deep learning models capable of integrating multimodal data have great potential for improving diagnostic accuracy and clinical decision-making. For example, combining information from MRI, CT, positron emission tomography (PET), and histopathological images can provide complementary insights into disease characteristics, treatment response, and patient outcomes [38]. Advanced fusion techniques, such as multi-input/multi-output networks and attention mechanisms, can facilitate the integration of multimodal data at different stages of the deep learning pipeline. By effectively capturing and fusing information from diverse sources, multimodal deep learning models can enhance feature representation, improve model robustness, and enable more accurate predictions. Future research may explore novel approaches for multimodal integration and fusion, with a focus on addressing challenges such as data heterogeneity, modality misalignment, and the semantic gap between modalities [38].

In addition to traditional convolutional and recurrent neural networks, future research in medical image analysis may explore advanced architectures, such as graph neural networks (GNNs) and capsule networks. GNNs are well suited for analyzing data with complex relational structures, such as connectivity graphs derived from anatomical or functional imaging data. By explicitly modeling the relationships between image elements (e.g., pixels and voxels), GNNs can capture spatial dependencies and contextual information, leading to more robust and interpretable representations [39].

Capsule networks, which are inspired by the hierarchical structure of the human visual system, offer a promising alternative to traditional CNNs for image representation and reasoning. Capsule networks encode information in the form of capsules, which represent the instantiation parameters of visual entities such as objects or parts. By preserving the spatial hierarchies and pose relationships between entities, capsule networks have the potential to improve generalization and robustness in medical image analysis tasks [40].

As deep learning models become increasingly complex and opaque, ensuring their interpretability and explainability is essential to gaining clinicians' trust and facilitating their clinical adoption. Interpretability refers to the ability to understand and interpret the decisions made by a model, whereas explainability involves providing human-understandable explanations for those decisions. In medical image analysis, interpretable and explainable deep learning models can help clinicians validate model predictions, understand underlying disease mechanisms, and guide treatment decisions [41]. Recurrent neural networks (RNNs), with their ability to capture temporal dependencies, have found applications in sequential data analysis within medical imaging. Notable examples include the interpretation of electrocardiograms (ECGs) for cardiac arrhythmia detection, where deep neural networks have achieved cardiologist-level performance. Furthermore, customized architectures and future directions in deep learning for medical image analysis offer promising avenues for innovation. Techniques such as attention mechanisms and GNNs are enhancing model performance, interpretability, and generalization capabilities. However, addressing challenges related to limited annotated datasets, model interpretability, and generalization across diverse patient populations remains paramount. Efforts towards efficient transfer learning, multimodal integration, and model interpretability are crucial for realizing the full potential of deep learning in revolutionizing healthcare delivery through improved diagnostic accuracy, personalized treatment strategies, and enhanced patient care.

Future research efforts should focus on developing techniques for model interpretability and explainability tailored to medical imaging tasks. These may include methods for visualizing model activations, attributing predictions to relevant image regions, and generating textual or graphical explanations for model decisions. By providing clinicians with transparent and interpretable insights into model behavior, interpretable and explainable deep learning models can foster trust, facilitate collaboration between humans and machines, and ultimately improve patient care.

## Conclusions

In summary, deep learning has emerged as a transformative force in medical image analysis, offering automated interpretation and precise diagnosis across various clinical domains. Despite its remarkable advancements, challenges persist, particularly in ensuring model interpretability, generalization, and robustness. However, the future holds immense promise, with continued interdisciplinary collaboration and innovation driving progress. By addressing existing challenges, deep learning has the potential to revolutionize healthcare delivery, improve diagnostic accuracy, and enhance patient outcomes. Through collaborative efforts and a commitment to excellence, deep learning stands poised to shape the future of medical imaging and diagnosis, ushering in an era of personalized and precision medicine.
